# Psychosocial Long-Term Effects of Young Adult Cancer Survivors: Study Protocol of the Longitudinal AYA-LE Long-Term Effects Study

**DOI:** 10.3389/fpsyg.2021.688142

**Published:** 2021-09-29

**Authors:** Kristina Geue, Anja Mehnert-Theuerkauf, Isabelle Stroske, Hannah Brock, Michael Friedrich, Katja Leuteritz

**Affiliations:** Department of Mental Health, Medical Psychology and Medical Sociology, University of Leipzig, Leipzig, Germany

**Keywords:** adolescent and young adult (AYA), cancer, psychological distress, quality of life, life situation

## Abstract

**Background:** About 3% of new cancer cases affect young adults aged between 15 and 39 years. The young age, the increasing incidence and the relatively good prognosis of this population lead to the growing importance to investigate the psychosocial long-term and late effects. The aims of the AYA-LE long-term effects study are: first, to assess the temporal course and related factors of life satisfaction and psychological distress of adolescent and young adult (AYA) cancer survivors; and second, to examine a specific topic in each of the yearly surveys in a more differentiated way.

**Methods:** This study represents a continuation of the longitudinal AYA-LE study. The existing sample of AYA cancer patients (t1: *N* = 577; t2: *N* = 514; aged between 18 and 39 years at diagnosis; all major tumor entities) was extended by four further survey points (t3: 2018, t4: 2019, t5: 2020, t6: 2021). In addition, a comparison sample of young adults without cancer was collected. We measured longitudinal data for outcomes such as quality of life, psychological distress, and fatigue with standardized questionnaires. Furthermore, each survey point included a different cross-sectional topic (e.g., health behavior, occupational situation, and compliance).

**Discussion:** The AYA-LE long-term effects study will show the long-term consequences of cancer in young adulthood. We expect at least complete data of 320 participants to be available after the sixth survey, which will be completed in 2021. This will provide a comprehensive and differentiated understanding of the life situation of young adults with cancer in Germany. The findings of our study enable a continuous improvement of the psychosocial care and specific survivorship programs for young cancer patients.

## Background

Around 3% of new cancer cases occur in young adults aged between 15 and 39 years (Gondos et al., 2013). This patient group is described by the National Cancer Institute as adolescent and young adults (AYA) (National Cancer Institute, 2019). Cancer in young adulthood is a non-normative life event (Brandstätter and Lindenberger, 2007) and often has significant physical, social and psychological consequences for those affected (Smith et al., 2016). Meanwhile, the complex developmental tasks that are typical of young adulthood (e.g., maturation of personality, entering into a partnership, starting a career, and starting a family and parenthood) have to be managed (Zebrack and Isaacson, 2012).

The young age, the increasing incidence in the last two decades (Smith et al., 2016; Fidler et al., 2017) and the above-average prognosis of this patient group increase the importance of questions of aftercare and long-term consequences for their care (Rabin et al., 2011). AYA survivors have an increased risk for the occurrence of long-term effects. In the 30 years following initial diagnosis, AYA are eight times more likely than their siblings to develop comorbidities such as cardiovascular disease (cardiotoxicity), endocrine and neurological disorders, diabetes or osteoporosis (Oeffinger et al., 2006). In addition, they have a two to three times higher risk of experiencing other cancers (AYA Oncology Progress Review Group, 2006; Hilgendorf et al., 2016).

The previous research findings have reported that AYA have a poorer quality of life and increased psychological stress when compared to their healthy peers (Quinn et al., 2015). In addition, it is known that young adults with cancer have high unmet supportive needs (Tsangaris et al., 2014). However, internationally, very few longitudinal studies have investigated the psychosocial situation of young adults with cancer.

Zebrack et al. (2014) interviewed patients up to a maximum of 16 months after diagnosis (t1: within 4 months after diagnosis; t2: 6 months after t1; t3: 12 months after t1) and showed that over one third of the participants reported a clinically significant level of distress at least once during the study period. Furthermore,12 months after diagnosis, 57% of the AYA patients expressed dissatisfaction with their level of information on at least one of the following topics: information on cancer itself, information on websites, infertility, physical activity or diet (Kwak et al., 2013). In their longitudinal study, Krüger et al. (2009) surveyed breast cancer patients up to 1 year after diagnosis. Compared to older patients, AYA patients had the lowest psychological burden but the relative differences to healthy peers were greater. Husson et al. (2017a,b) investigated *n* = 176 AYA patients with regard to their quality of life and post-traumatic growth at three points in the first 2 years after diagnosis. In terms of quality of life, the greatest improvement was seen in the first 12 months after cancer diagnosis. However, the quality of life of AYA patients compared to the age-matched general population was also significantly lower after 2 years. Furthermore, Chan et al. (2018) published a longitudinal study from the Asian region that examined the distress of AYA patients (*N* = 65) in the first 6 months after diagnosis. They showed that there was a decrease in distress from the time of diagnosis to 6 months later.

The few research findings to date regarding the psychosocial life situation of young adults with cancer are not sufficient to establish specific, comprehensive and structured follow-up concepts. Smith et al. (2019) emphasize the importance of the examination of late effects and psychosocial outcomes for the care of young cancer patients. In addition, many aspects of what is known about AYA have been deduced from long-term pediatric cancer survivors (Warner et al., 2016). The aim of the continuation of the study is: first, to assess the temporal course and related factors of life satisfaction and psychological distress of AYA cancer survivors; and second, in each of the yearly surveys, a specific topic of life will be examined in more detail in a cross-sectional design.

Based on the longitudinal study presented here with research-based data on essential life domains (e.g., occupational situation, health behavior, social relationship) of the AYA, urgently needed evidence-based survivorship programs can be designed and established.

## Research Questions

The primary research questions follow:

1. What changes occur over time in regard to life satisfaction and psychological distress?
2. What other factors (e.g., sociodemographic, medical, and psychosocial) are associated with time changes in life satisfaction and psychological distress?
3. What differences exist between young adults with cancer compared to healthy peers in life satisfaction and psychological distress?

The selected further research questions follow:

4. How does the health behavior (e.g., nicotine consumption, alcohol consumption, use of illegal drugs, physical activity, nutrition, and adherence) of young adults with cancer compare to that of healthy people?
5. How does the current occupational situation (e.g., employment status, working hours per week, cognitive and physical performance, and income) of young adults with cancer change over time? And, how does it compare with that of healthy people?

## Methods

### Study Design

This study represents a continuation of the previous study “Life satisfaction, care situation and support needs of cancer patients in young adulthood” (AYA-LE study) (Leuteritz et al., 2017). This study is implemented as a prospective longitudinal survey, which extends the previous study with two measurement points (t1 and t2) by at least four further annual points in time (t3: 2018, t4: 2019, t5: 2020, t6: 2021). In addition, a comparison group of healthy young adults was surveyed at measurement time t3 and t4.

**Figure 1:**
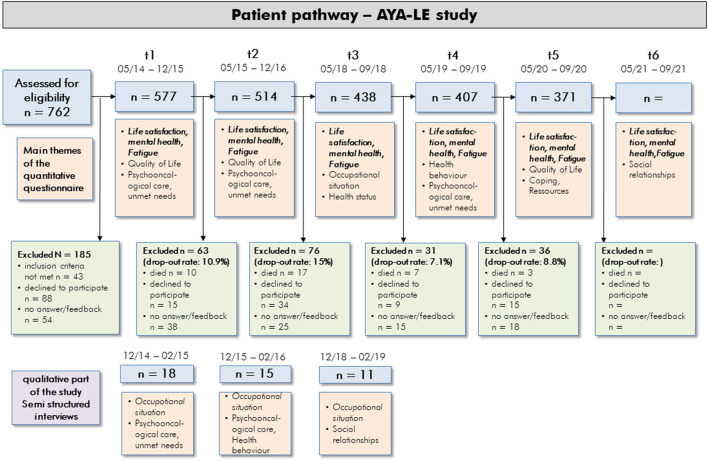
Flowchart of the AYA-Le study participants and main survey themes.

Semi-structured interviews were conducted at measurement time point's t1, t2, and t3, each repeated with the same interviewees and analyzed with Mayring's qualitative structuring content analysis. More detailed information on the measurement time points can be seen in Figure 1. The complete study was funded by the German Cancer Aid.

### Participants

#### AYA Patients: Inclusion Criteria, Recruitment, and Data Collection

The patients must meet the following inclusion criteria:

1. Age at diagnosis: 18–39 years. (Our study aims to draw conclusions about patients treated in adult oncology departments. In Germany, patients younger than 18 years are treated in pediatric oncology units, and patients over 18 are treated in adult oncology units. This is the reason why we focused on 18–39 year olds).
2. First manifestation of cancer (all malignant tumor identities C00-C97).
3. Diagnosed within the last 4 years at t1.
4. Participation of the t1 and t2 survey.

AYAs are excluded from study participation if an initial screening of their data reveals that:

- They are unable to speak German;
- They are physically or cognitively unable to participate in the survey; or
- They did not provide written consent.

The recruitment in the AYA-LE study was managed in cooperation with four rehabilitations clinics, two tumor registries and 16 acute care hospitals. In May 2018, the 514 patients of the existing sample received an email link to answer the third study questionnaire (t3). The patients are already familiar with this procedure thanks to the first two survey time points (t1, t2). As in the previous study, the online questionnaire was created using the LimeSurvey software. If no email address was available or requested, then the participants received the questionnaire and documents by post.

Reminders were mainly sent by email continuously every 10 days. Approximately 4 weeks after the t3 invitation, patients who had not yet responded received a first postal reminder with questionnaire and participant documents in paper form. The second postal reminder took place 3 weeks later. Address data that were no longer up to date were updated at the residents' registration office. This procedure is now repeated for the yearly surveys, always including all of the participants of the last survey.

#### Comparison Group: Inclusion Criteria, Recruitment, and Data Collection

The inclusion criteria for the comparative sample were: women and men from the general population who had not had cancer were selected from the general population according to the age and gender of the patient sample at t2. The same exclusion criteria as for the AYA patients were applied.

For the formation of the comparative sample, group information was provided to the Leipzig Register of Residents to obtain a randomized list for men and women from the general population of Leipzig between 18 and 45 years of age. This requirement represented the average age and gender distribution of the AYA patient sample at t2. Taking recruitment drop outs into account, about three times as many people as the AYA sample at t2 (*N* = 514) were requested (*N* = 1,598).

To keep the time required to fill out the study questionnaire reasonable, the survey was divided into two points in time with intervals of 12 months. The two exclusively cross-sectional study questionnaires each contain different topics (occupational situation, health behavior at the same time as the patient survey). The instruments used for the comparison group are marked with * in Table 1. The comparison sample was contacted by post once within 2 months (January to February 2018) in three waves. The second part of the survey of the comparison group was carried out 12 months later in January 2019. Within this survey, two postal reminders were sent out 3 and 6 weeks after the respective questionnaire was sent out.

**Table 1:** Overview of study instruments used from t1 to t6.

**Instruments**	**t1**	**t2**	**t3**	**t4**	**t5**	**t6**
**BASIC VARIABLES (PSYCHOLOGICAL DISTRESS, LIFE SATISFACTION, FATIGUE)**						
**Hospital Anxiety and Depression Scale (HADS; Herrmann et al.**, **1995**)^*^The HADS is a measuring instrument for anxiety and depression that is specially geared to people with somatic diseases. The 14 items can be rated on a 4-point Likert-Scale. High scores (max. 21) implicate high values of anxiety/depression.	X	X	X	X	X	X
**Distress Thermometer (Mehnert et al.**, **2006**)^*^The Distress Thermometer is the German version of the screening-tool for psychosocial distress in cancer patients, initially developed by the National Comprehensive Cancer Network (NCCN). It contains a single-item-scale from 0 to 10 for perceived psychosocial distress in the last week (and a list of problems to tick whether these problems occurred or not). A score of more than four indicates psychosocial distress.	X	X	X	X	X	X
**Questionnaire of life satisfaction (FLZ-M; Henrich and Herschbach**, **2000**)^*^The FLZ-M is used to measure the subjective life satisfaction and its relative importance in different areas of life on a 5-point Likert-Scale. The Questionnaire focusses upon the two modules “global life satisfaction” and “satisfaction in health,” with eight items each and one overall-item.	X	X	X	X	X	X
**Questions about Sexuality (Questionnaire of life satisfaction uesexuality scale, FLZ-M; Henrich and Herschbach**, **2000**)^*^As 1 of 10 subscales of the FLZ-M (measuring life satisfaction), the sexuality scale is used to quantify sexual satisfaction considering physical attraction, sexual efficiency, sexual contacts, and sexual reactions. 7 items are used to rate sexual satisfaction on a 7-point Likert-Scale from “very unsatisfied” (1) to “very satisfied” (7).	X	X	X			X
**Perceived Adjustment to Chronic Illness Scale (PACIS; Hürny et al.**, **1993****)**PACIS is a single-item measure for coping with chronic illness. On a scale of 1 (none) to 100 (a great deal) it states the subjective effort to cope with chronic illness.	X	X	X		X	X
**Fatigue Module (EORTC-QLQ-FA-12; Weis et al.**, **2017**)^*^This Questionnaire assesses physical, emotional, and cognitive fatigue in cancer patients with 10 items that can be rated on a 4-point scale. Higher scores indicate high levels of fatigue. Additional two items measure the influence of fatigue on daily activities and social live.	X	X	X		X	X
**Quality of life (EORTC QLQ-C30; Schumacher et al.**, **2003**)^*^The EORTC QLQ-C30 consists of 30 items and is used to measure cancer patients' quality of life on five function scales (physical, role, emotional, cognitive, and social), three symptom scales (fatigue, nausea, pain), six symptom scales (e.g., dyspnea, insomnia, financial difficulties), and a two-item global health status/QoL scale.	X	X		X		
**Late adolescence and young adulthood survivorship-related quality of life measure (LAYA-SRQL; Richter et al.**, **2018**)^*^The LAYA-SRQL is an instrument for assessing health-related quality of life in AYA cancer patients. We used a shortened version of the LAYA-SRQL and participants rated 15 items (range 1 to 4) for both satisfaction and impact.			X			
**Basic Psychological Need Satisfaction Scale (BPNS; Gagné**, **2003**)^*^The Basic Psychological Need Satisfaction is a self-report instrument assessing the need satisfaction in general. It has 21 items assessing the three basic psychological needs of autonomy, competence, and relatedness defined by self-determination theory.			X			
**Brief Pain Inventory (BPI; Cleeland and Ryan**, **1991****; Radbruch et al.**, **1999**)^*^The Brief Pain Inventory (BPI) is a simple tool to assess the severity of pain and its impact on common dimensions of feeling and function. We used 13 items from the BPI with 10-point Likert-scales indicating the intensity of pain in general, at its worst, at its least, and right now.			X			
Questions about life attitudes	X	X				
Sociodemographic variables^*^(partnership, children, education, housing situation, critical life events or changes since last survey)	X	X	X	X	X	X
**Disease-related variables**(changes regarding treatment, comorbidities or cancer diagnosis in the past for healthy control group)	X	X	X	X	X	X
**PSYCHOSOCIAL CARE (MAIN TOPIC T1 AND T2)**						
**Illness-Specific Social Support Scale (Soziale UntersttUnter bei Krankheit raSSUK-8; Ullrich and Mehnert**, **2010****)**The SSUK-8 quantifies the participant's perception of social support of subjectively important relationships by rating how often these important persons show certain (un)helpful behaviors. It consists of 8 items, to be rated on a range from 0 (never) to 4 (always).	X	X				X
**Short Form Supportive Care Needs Survey Questionnaire (SCNS-SF34-G; Lehmann et al.**, **2012****)**On a 5-point Likert-Scale from 1 (no need, not applicable) to 5 (high need), 34 items are used to measure the perceived care needs of cancer patients. The Questionnaire focuses on type and amount of support they might need in five domains (health system and information, psychological state, physical and daily living, patient care and support, and sexuality needs).	X	X		X		X
**Global Motivation Scale (GMS; Sharp et al.**, **2003**)^*^The 18-item Global Motivation Scale (GMS) assesses six different types of motivation: (1) intrinsic motivation; extrinsic motivation: (2) integrated, (3) identified, (4) introjected and (5) external regulation) and (6) a motivation as described by Self-Determination-Theory. The subscale for intrinsic motivation consists of three items distinguishing between intrinsic motivation toward knowledge, stimulation and accomplishment. The Global	X	X	X			
**F-SozU (German Social Support Questionnaire; Sommer and Fydrich**, **1989**)^*^The questionnaire measures social support and satisfaction with that social support from the respondent's perspective. The short form consists of 14 items.				X		
Utilization, satisfaction and evaluation of psychosocial care^*^ (psychological counseling, social-legal counseling and other psychosocial care) as well as needs and preferences regarding psychosocial care services	X	X				X
**OCCUPATIONAL SITUATION (MAIN TOPIC T3)**						
**Work Ability Index – short version (WAI-r; Tuomi et al.**, **1998****; Ilmarinen**, **2009****; Bethge et al.**, **2012**)^*^Using seven items, the WAI-r assesses the work ability of employed individuals with respect to personal resources and working condition (Zwart et al., 2002).	X	X	X			
**Questions on the Importance of Work (Bürger et al.**, **2001****; Bürger**, **2004****; Mehnert and Koch**, **2013**)^*^This questionnaire is a measurement for the reintegration to work after a disease considering the personal relevance of working activities as well as employers and others (e.g., family, physician) view of the patients employment.			X			
**Questions about professional adjustments** (self-developed items assessing the individual professional adjustments, e.g. reduction of weekly working hours, progressive reintegration, workplace health promotion, and the helpfulness of these adjustments)			X			
**Copenhagen Psychosocial Questionnaire (COPSOQ; Kristensen et al.**, **2005****; Nübling et al.**, **2006**)^*^The COPSOQ quantifies the subjective extent of psychosocial workload. Four items of the COPSOQ were used to measure the extent of cognitive impairments with a five-point Likert-Scale. Participants had to rate from “always” (1) to “never/hardly ever” (5) how often problems in concentrating, decision-making, remembering and clear-thinking occurred during the last week.	X	X	X			
Occupational situation^*^(Employment status, time of sick leave, characterization of work, weekly working hours)	X	X	X		X	X
Financial situation^*^(Monthly household net income)	X	X	X			
**HEALTH BEHAVIOR (MAIN TOPIC T4)**						
**World Health Organization Alcohol, Smoking and Substance Involvement Screening-Test (WHO ASSIST; Schütz et al.**, **2005****; Humeniuk et al.**, **2008**)^*^The German version of the WHO ASSIST measures addictive drug consumption taking the 10 most used drugs (alcohol, tobacco, cannabis, and cocaine etc.) into account. It contains 71 items with consumption-relevant questions (frequency, craving, consumption induced problems etc.) for each drug.				X		
**Multiple health behaviour (MHB-39; Wiesmann et al.**, **2003**)^*^The questionnaire Multiple Health Behavior (MHB-39) is an instrument designed for assessing habitual health-related behavior. We chose 15 items based on their content and statistical relevance to our study. The items are assigned to six different categories (active lifestyle, medical compliance, substance avoidance, personal safety-related behavior, nutrition, and hygiene).	X	X				
**The Exercise and Sports Activity Questionnaire (BSA-F; Fuchs et al.**, **2015**)^*^The BSA-F measures the daily exercise and sports activity during the last 4 weeks in three different parts, considering physical activity during working life (3 items) as well as activity in leisure time (9 items) and sports (1 item). Participants must state frequency, duration, and type of each activity.				X		
**Food Frequency List (FFL; Winkler and Döring**, **1998**)^*^The FFL is a list of 24 different food items. Participants must state how often they use the suggested food on a scale from 6 (almost daily) to 1 (never) without a given timeframe.				X		
**Pittsburgh Sleep Quality Index (PSQI; Buysse et al.**, **1989****; Backhaus et al.**, **2002**)^*^Composed of 19 items, the PSQI measures the subjective sleep quality and disturbances over the last month. It focuses on the seven sleep-components: quality, latency, duration, efficiency, disturbances, sleeping medication and daily tiredness.				X		
**General Self-efficacy Short Scale (ASKU; Beierlein et al.**, **2017**)^*^Three items with a 5-point scale are used to measure the general self-efficacy that is the subjective ability to plan and realize actions for the purpose of achieving a desired goal.				X		
Relaxation, Body weight, Compliance, changes since cancer diagnosi^*^(Self-developed items assessing the individual ability to relax and irritability, adherence to medical advice, questions about body weight, conducive and hindering factors for sleep, nutrition, physical activity and medical advice.)				X		
**COPING AND RESSOURCES (MAIN TOPIC T5)**						
**Generalized Self-Efficacy Scale (SWE; Schwarzer and Jerusalem**, **1999****; Hinz et al.**, **2006****)**The SWE measures the perceived self-efficacy (i.e., one's own perceived ability to master (daily) severe challenges) on a scale of 10 four-level items. High scores implicate high perceived competences to cope with problems in daily life.					X	
**Cancer Behavior Inventory – brief version (CBI-B; Heitzmann et al.**, **2011****)** The CBI-B was used to assess self-efficacy especially for coping with cancer. Each of the 12 items represent one of the four domains of potential coping behavior: Maintaining Independence and Positive Attitude, Participating in Medical Care, Coping and Stress Management and Managing Affect. On a 9-point Likert-Scale, participants can rate how confident they would feel while performing a suggested coping strategy.					X	
**Resilience Scale (Schumacher et al.**, **2005****)**This scale is used to measure psychosocial stress-resistance. The short version consists of 11 items that can be answered on a 7-level scale from 1 (Disagree) to 7 (Agree).					X	
**Stress-Related Growth Scale (German version: Persönliche Reifung nach Belastungen; Maercker and Langner**, **2001****)**This Scale quantifies personal growth after stressful life-events in terms of positive changes after such an event. It contains 15 items about changes regarding different aspects in life because of mentioned life-event stress to be rated on a 3-point scale from 0 (not at all) to 2 (a great deal).					X	
**Demoralization Scale (Kissane et al.**, **2004****)**Regarding the five factors loss of meaning, dysphoria, disheartenment, helplessness, and sense of failure, the Demoralization Scale measures demoralization as one expression of existential stress. On a 5-point Likert-Scale, 24 items must be answered. Higher levels of demoralization are expressed by higher scores.					X	X
**Sense of Coherence Scale (Singer and Brähler**, **2007****)**The short version of the Sense of Coherence Scale consists of 9 items to be rated on a 7-point answer scale. Defined by Antonovsky (1979), Sense of Coherence describes the ability of a person to perceive the environment and one's own challenges/experiences as predictable, explicable, meaningful, worthy, and manageable.					X	
**Locus of Control Survey (German: Fragebogen zur Erhebung von Kontrollüberzeugungen – KKG; Lohaus and Schmitt**, **1989****)**The KKG measures locus of control based on health and illness, considering the three dimensions: Belief that health is controllable by oneself, by others, as well as that health is dependent of coincidences or fate. There are seven items for each dimension, to be answered on a 6-point Likert-Scale.					X	
**Acceptance and Action Questionnaire (AAQ; Bond et al.**, **2011****)**The AAQ examines one's handling of unpleasant thoughts and feelings referring to psychological inflexibility, acceptance, and experiential avoidance. It consists of 7 items to be rated on a 7-level scale.					X	
**Short Version of the Freiburg Questionnaire on Processing of Illness (Freiburger Fragebogen zur Krankheitsverarbeitung – FKV-LIS; Muthny**, **1989****)**To measure different behaviors for processing of somatic illness, the FKV-LIS can be used. 35 items on a 5-point Likert-Scale capture different ways of processing on a cognitive, emotional, and behavioral level.					X	
**European Health Literacy Questionnaire (HLS-EU-Q16; Röthlin et al.**, **2013** **−>** **GK1-16)**The HLS-EU-Q16 measures the four dimensions of health literacy (accessing, understanding, assessing and applying health information) in the areas of disease prevention, health promotion and health care.					X	
**Feelings**(two self-developed items assessing current feelings about dealing with the disease and its consequences)					X	
**SOCIAL RELATIONSHIPS (MAIN TOPIC T6)**						
**Social Impact Scale (SIS; Eichhorn et al.**, **2015****)**Considering four subscales (social isolation, social rejection, internalized shame, and financial insecurity), the SIS measures the perceived stigmatization in cancer patients using 24 items on a 4-level scale.						X
**Marital Quality Questionnaire (Partnerschaftsfragebogen – PFB; Hahlweg**, **1979****)**The PFB measures the marital quality focussing on three dimensions named tenderness, conflict behavior, and community/communication. Participants are supposed to rate in 10 items whether their partner is showing certain behavior “never/almost never” (0) to “quite often” (3).						X
**Experiences in Close Relationship-Revised (ECR-RD; Ehrenthal et al.**, **2009****)**To capture attachment in adults, the ECR-RD is used, focusing on two dimensions: attachment-related anxiety and avoidance in one's general experiences in partnerships. Each dimension is composed of 18 items to be answered on a 7-level scale.						X
**Patient Reactions Assessment (PRA; Brenk-Franz et al.**, **2016****)**The PRA quantifies the subjective quality of the physician-patient relationship as assessed by the patient. 15 items are divided into the three subscales information, communication and affectivity to be rated on a 7-point Likert-Scale.						X

* *instruments were also used in the survey of healthy young adults (comparison group)*.

To increase the participation rate, all of the study participants (AYA and comparison group) received a compensation fee of 10 Euros for completing the questionnaire at each measuring point.

### Study Measures

The longitudinal survey included standardized questionnaires on psychological distress (HADS, distress thermometer), life satisfaction (questionnaire on life satisfaction) and fatigue (EORTC-QLQ FA-12) (which we already used from t1 to t6). The basis for the selection of these measurement instruments was their established use in psychooncology.

In addition, we used standardized questionnaires and self-developed items to assess cross-sectional topics like occupational situation (t3), health behavior (t4), coping resources (t5), and social relationships (t6). For this purpose, standard measurement instruments from the German-speaking world were selected, which, if possible, have already been used and tested in psycho-oncological studies.

All used instruments shown and described in Table 1.

### Data Analyses

We use the Statistical Package for the Social Sciences 23 (IBM SPSS Statistics) for all of the quantitative statistical data analyses. The data collected in this study will be described in terms of mean, standard deviation, median, minimum and maximum. Frequencies of the primary outcomes psychological distress and QoL reported in combination with confidence intervals. Histograms and boxplots will be used for graphical representation.

The primarily exploratory evaluation of the AYA-LE-study includes questions on descriptive expressions, changes over time, influencing factors in the cross-section and longitudinally, and comparative analyses between AYA and the comparison group.

To compare the two groups with respect to a continuous outcome variable, *t*-tests (in case of normal distribution) or Mann-Whitney-*U*-tests (in case of non-existence of a normal distribution) are applied. To compare the two groups with respect to a categorical outcome variable, crosstabs with chi-square tests and (if necessary) generalized exact Fisher-tests are calculated. When comparing two measurement points, the *t*-test or the Wilcoxon test for paired samples, supplemented by crosstabs and possibly the Mc-Nemar test are performed. When comparing multiple measurement points, (co-)variance analyses for repeated measures are performed, or the Friedmann test if the normal distribution assumption is not given.

For multivariate analyses with metrically scaled outcome variables, multiple linear regressions are calculated or logistic regressions are used for binary target variables. The impact of age, gender, diagnosis groups and treatment are to be investigated. The analyses will be preceded by explorative variance analyses. If the identified influencing variables show different (inconsistent) effects for patients and the healthy control group, then additional moderation analyses will be performed to identify possible interaction effects (as moderator the group variable “AYA” vs. comparison group investigated).

## Results

### AYA Patients

At the first survey (t1), a total of 577 young cancer patients were included in the study. Recruitment details are outlined in the study protocol that was published in the previous study (Leuteritz et al., 2017). Of the 577 t1 participants, 35.7% (*N*=206) withdrew from the study by the time of the t5 survey. These drop outs consisted of deceased, active rejects, and unreached patients (Figure 1). Thus, at t5 371 complete datasets from t1 to t5 of the AYA patients exists. For t6, we also expect a response rate of approx. 90%.

### Comparison Group

Of the *N* = 1,598 Leipzig people who were contacted, *n* = 421 responded, of which *n* = 15 did not meet the inclusion criteria. *N* = 1,177 (74.4%) people did not answer or refused to be interviewed (non-responder). Thus *n* = 406 (25.5%) respondents were present in the first survey. Of these, *n* = 372 respondents also filled out the second survey with the focus on health behavior. The sociodemographic and medical characteristics of the samples are shown in Table 2.

**Table 2:** t4-Follow-up characteristics of the patient sample (AYA = 407) and the comparison group (CG = 406).

	**AYA cancer patients (*N* = 407)**	**Young adults – comparision group (*N* = 406)**
**Age at survey time Mean (SD)**	34,76 (SD 6.31)	32.32 (SD 5.69)
**Sex: female**	306 (75.2%)	230 (61.8%)
**Children: (yes)**	164 (40.3%)	148 (39.8)
**Relationship: (yes)**	324 (79.6%)	287 (77.2%)
**Housing situation:**		
By oneself	104 (25.6%)	71 (19.1%)
With partner	267 (65.6%)	243 (65.3%)
Flat share	17 (4.2%)	39 (10.5%)
With parents	19 (4.7%)	9 (2.4%)
**Highest educational degree:**		
Secondary educational degree (10 years)	147 (36.1%)	99 (27.0%)
Highschool degree (>10 years)	249 (61.2%)	265 (72.2%)
**Cancer diagnosis**		
Solid	277 (68.1%)	
Hematological	130 (31.9%)	
**Time since diagnosis in months**		
Mean (SD); min-max:	56 (SD 9.11) 30.6–91.95	

## Discussion

The continuation of the existing AYA-LE study *via* several additional surveys illustrates the long-term consequences of cancer in young adulthood. A major strength of this study is the collection of longitudinal data of the outcome's quality of life, psychological distress, and fatigue over a very long period of time (at least 6 years). We expect at least 320 complete datasets to be available after the sixth survey, which will be completed in September 2021. To our knowledge, the existing AYA longitudinal studies (Krüger et al., 2009; Kwak et al., 2013; Zebrack et al., 2014) cover a significantly shorter period of time. Furthermore, a different cross-sectional thematic focus is examined at each measurement point. This creates comprehensive and differentiated findings of the life situation of young adults with cancer in Germany. In addition, the questioning of young adults without cancer in the continuation of the AYA-LE study will enable a comparison in psychological distress, quality of life, occupational situation and the health behavior between AYA patients and young adults without cancer.

The low dropout rate over the measurement points is to be emphasized. This can be attributed to the high interest of the patients, who wish to be addressed as a special patient group. To do justice to this wish, the sponsor of the study (German Cancer Aid) is producing the first guide for young adults with cancer in Germany with the support of our AYA research group. Through various strategies (e.g., Christmas and Easter greetings, and multiple reminders) we intend to create a high-rate of participation in the longitudinal study. The resulting large sample size allows different subgroup analyses.

In the first study period (t1, t2), approximately every second participant reported clinically increased anxiety, indicating a significant need for support. Critical life areas were financial and occupational situation as well as family planning and sexuality. Patients with a low net household income, another illness or without social support are particularly at risk of losing life satisfaction in the short and long term. There was a unmeet need with regard to psychological issues, the desire to have children and sexuality. At both times of the survey, the most frequent wishes were for general or psychological counseling, for age-appropriate sports activities and relaxation procedures, and for socio-legal/occupational counseling.

For the further results in the longitudinal study, we expect a stabilization of life satisfaction with a continuing high level of anxiety. With regard to the cross-sectional topics of occupational situation, health behavior, social relationship and coping resources, which have only been marginally investigated so far, we hope to gain a first detailed and differentiated insight in order to design appropriate intervention concepts.

## Data Availability Statement

The raw data supporting the conclusions of this article will be made available by the authors, without undue reservation.

## Ethics Statement

The studies involving human participants were reviewed and approved by University of Leipzig. The patients/participants provided their written informed consent to participate in this study.

## Author Contributions

This study's design and assessments were conceptualized and developed by KG, KL, and AM-T. The implementation and conduct of the study was coordinated by KG, KL, IS, MF, and HB. KG and KL wrote an outline of the paper, which was carefully revised, edited and discussed by AM-T, IS, HB, and MF. All of the authors read and approved the final manuscript.

## Funding

This study was funded by a grant from the German Cancer Aid (Grant No: 70112752 and 70113932). The funding bodies played no role in the design of the study and collection, analysis, and interpretation of data and in writing the manuscript.

## Conflict of Interest

The authors declare that the research was conducted in the absence of any commercial or financial relationships that could be construed as a potential conflict of interest.

## Publisher's Note

All claims expressed in this article are solely those of the authors and do not necessarily represent those of their affiliated organizations, or those of the publisher, the editors and the reviewers. Any product that may be evaluated in this article, or claim that may be made by its manufacturer, is not guaranteed or endorsed by the publisher.

## Abbreviations

AYA: Adolescent and Young Adult
QoL: Quality of life; IBM SPSS Statistics.
